# Editorial: Plant and fungal metabolites: innovations in special dietary foods and disease prevention

**DOI:** 10.3389/fnut.2026.1797174

**Published:** 2026-02-24

**Authors:** Paucean Adriana

**Affiliations:** Department of Food Engineering, Faculty of Food Science and Technology, University of Agricultural Sciences and Veterinary Medicine, Cluj-Napoca, Romania

**Keywords:** fungi, healthcare, nutraceuticals, plants, secondary metabolites

As natural products become central to modern healthcare, the need for comprehensive databases and advanced analytical tools (e.g., bioinformatics, metabolomics) has increased. Plants and fungi produce a wide range of secondary metabolites, including polyphenols, flavonoids, terpenoids, and polysaccharides. Although these secondary metabolites are not essential nutrients, they have a significant influence on oxidative stress, inflammation, and metabolic health. This growing body of knowledge improves the understanding of how plants and fungi represent alternative sources of nutritional compounds and nutraceutical products, offering novel ways to mitigate health problems. Consequently, food is increasingly viewed not merely as an energy source, but as a complex system critical for disease prevention.

The collection of publications presented in this Research Topic covers a wide spectrum of approaches to study the chemical and biological characteristics of plant and fungal metabolites that could be used to prevent diseases.

Zhang et al. provided an extensive review outlining the current literature regarding polysaccharides derived from *Ophiopogon japonicus*, including methods of extraction and purification, structural features, and pharmacological effects. The article discusses the hypoglycemic, cardioprotective, immunomodulatory, antioxidant, and anti-inflammatory effects of polysaccharides, and it is important to note that the relationship between polysaccharide structure and function plays a crucial role. Additionally, the authors demonstrated that chemical modifications can enhance polysaccharide bioactivity, bioavailability, and other characteristics, thus emphasinzing the necessity of rational design approaches when polysaccharides are intended for use in functional foods and dietary supplements are developed. Unfortunately, there are still gaps in knowledge regarding the mechanism of action and clinical validation before these compounds can be applied.

Phenolic compounds are key elements among plants and fungal metabolites. Metabolomics approaches have recently pointed out unprecedented insight into the complexity of phenolic mixtures and how they can be associated with biological activity. She et al. describes the phenolic profile of *Camellia oleifera* oil using targeted metabolomics and statistical models. Their results indicate that this oil has a surprisingly rich phenolic composition that is largely due to flavonoid compounds and that strong correlations exist between certain phenolic compounds and antioxidant activities. Furthermore, they identified critical metabolic pathways and phenolic compounds associated with antioxidant activities and provide useful information about optimizing extraction and developing products based on camellia oils that are designed for health and specialized dietary use.

Guo et al. aimed to identify the geographical origins of *Codonopsis Radix* from different origins based on odor information. Quality assurance is critical given the variability caused by geography and processing. By using chromatographic fingerprinting, electronic nose technology for analyzing volatile compounds, and OPLS-DA statistical analysis, the authors showed that the information regarding odors, especially the presence of certain volatile compounds, such as hexanal, can be used to identify samples based on their geographical origin. Their work may potentially lead to the development of rapid, portable, and green identification tools, which need to be developed due to expanding markets and globalization of supply chains for functional plant-based ingredients.

Chen et al. investigated the molecular mechanisms of Huanglian Wendan Decoction (HLWD) in treating type 2 diabetes mellitus-associated skeletal muscle lesions. Combining bioinformatics and experimental validation, they identified specific apoptotic and immune pathways targeted by HLWD. These findings clarify the compound's mechanism of action, supporting the integration of traditional remedies into modern therapy pending clinical validation.

Alam et al. conducted a chemico-pharmacological analysis of *Colocasia affinis* Schott. Using *in vitro, in vivo* and computational docking studies, they identified distinct activities in specific extracts. Thus, the aqueous fraction exhibited antioxidant and antimicrobial properties, while the dichloromethane fraction showed antidiarrheal and analgesic effects. This underscores the potential of vegetables such as dwarf elephant ear as rich sources of bioactive, drug-like compounds.

Pan et al. studied *Ganoderma lucidum* (well known as a medicinal mushroom) to evaluate the triterpenoid profiles of a traditionally cultivated mushroom vs. an artificially selected/trained mushroom to produce a new variety with 50% higher content of triterpenoids, thereby enhancing the antioxidant capacity of the new strain. Thus, the novel strain exhibits evidence not only of a higher content of chemical constituents to support the increased functional benefits from this medicinal mushroom but also has advantages to enable better cultivation/standardization and therapeutic benefit from mushroom dietary products. Optimizing strains of mushrooms through processing techniques will ensure that mushroom-based dietary products are manufactured with consistent pharmacological effects.

In conclusion, by providing a wide-ranging survey of both traditional and novel approaches to dietary supplement and nutraceutical research, the studies included in this Research Topic offer a composite overview of recent advances in the field. These contributions offer new insights into how dietary phytochemicals support health promotion by linking nutrition to pharmacology and food technology. This Research Topic will foster interdisciplinary research and the development of innovative, scientifically validated functional foods to help address the increasing global burden of non-communicable diseases.

